# Decision-making criteria for damage control surgery in Japan

**DOI:** 10.1038/s41598-019-51436-x

**Published:** 2019-10-17

**Authors:** Nao Urushibata, Kiyoshi Murata, Yasuhiro Otomo

**Affiliations:** 1 Emergency Medicine and Acute Care Surgery, Matsudo City General Hospital, 993-1 Sendabori, Matsudo, Chiba 270-2252 Japan; 2 Trauma and Acute Critical Care Center, Tokyo Medical and Dental University Hospital of Medicine, 1-5-45 Yushima, Bunkyo-ku, Tokyo 113-8510 Japan

**Keywords:** Medical research, Risk factors

## Abstract

Controversy still remains regarding the optimal criteria for selecting damage control surgery (DCS). Our objective was to propose an indication for implementing DCS for abdominal trauma requiring emergency laparotomy. This was a multicenter, retrospective, observational study that used data from the Japan Trauma Data Bank. Patients who underwent emergency laparotomy were included. We compared the patients regarding the performance of DCS. Of the 4447 patients included in the study, 532 patients were in the DCS group and 3915 patients were in the non-DCS group. Logistic regression analysis revealed that body temperature, level of consciousness (Glasgow Coma Scale), and type of injury (blunt or penetrating) were independent predictors of DCS. Using these predictors, we created the Damage Control Indication Detecting score. The score showed a positive correlation with mortality. The score was obtained as 5 of 9 points in total, revealing mortality of 30.8%, sensitivity of 64.8%, and specificity of 70.0%. The area under the curve for the receiver operating characteristic curve was 0.715. This score can help surgeons determine when to perform DCS. However, more than 95% of trauma cases in Japan involve blunt injuries, suggesting that the results of our study may not be applicable internationally.

## Introduction

Hypothermia, coagulopathy, and acidosis are widely known as the “deadly triad,” which describes the severity of physiological damage in a trauma patient^1,2^. In severe trauma patients, emergency surgery involves damage control surgery (DCS), which is implemented as a rescue attempt as conventional surgery could be harmful^3^. Although the legitimate criteria for selecting DCS remain unclear, many previous studies used the “deadly triad” as an indicator of DCS. Although its specificity is considered to be fairly high, its sensitivity is not high enough to be universally accepted as a DCS indicator. As such, we sought to establish new criteria for DCS, paying particular attention to prehospital patient data.

## Objective

Our objective was to propose an indication for implementing DCS in patients with severe abdominal trauma who require emergency surgery.

## Methods

### Data source

This retrospective, observational study used data from the Japan Trauma Data Bank (JTDB). This database is a nationwide trauma registry established in 2003 by the Japanese Association of Trauma Surgery and the Japanese Association for Acute Medicine to improve the quality of trauma care in Japan. In 2015, 254 emergency hospitals voluntarily participated in this registry, most of which (90%) are tertiary-level emergency hospitals that have a role equivalent to level 1 trauma centers in other countries.

At these participating facilities, patient data are collected using web-based systems after anonymization, and the data are managed by the Association for Japan Trauma Care and Research. The database contains patients’ demographic data; vital signs on arrival, including systolic blood pressure (SBP), heart rate (HR), and Glasgow Coma Scale (GCS); type of trauma (blunt or penetrating); severity of injury using the Injury Severity Score (ISS); performance of Focused Assessment with Sonography in Trauma (FAST); in-hospital procedures with indications and time of the procedure; and status at discharge of each patient. Types of blunt trauma included in the JTDB registry are as follows: motor vehicle accident (car, bike, bicycle, pedestrian, and other), fall (stairs, stumble, and free fall), injury by machine (rotary, press, and other), injury by falling object, injury by explosion, press injury (heavy object and collapse), train, sports, and others. Types of penetrating trauma included in the JTDB registry are stab, gunshot, impalement injury, and others. In-hospital procedures included abdominal surgeries and the specific type of surgery performed. Type of emergency abdominal surgery included in the JTDB registry include liver (suture and resection), spleen (suture and resection), kidney (suture and resection), pancreas (suture and resection), pancreatoduodenectomy, stomach, duodenum, small intestine, large intestine, colostomy, cholecystectomy, bladder suture, urostomy, urinary reconstruction, vascular surgery, DCS, and others. Trauma patients with an abbreviated injury scale (AIS) of ≥3 are registered in the JTDB^4,5^.

### Sample population

We included trauma patients who were registered in the JTDB from January 2004 to December 2014. We extracted patients who underwent emergency abdominal surgery and excluded burn victims, patients with an ISS of 75, and patients who did not have any type of emergency abdominal surgery. We divided the selected patients into two groups: DCS and non-DCS. We defined the DCS group as patients who were registered as having undergone DCS in the database. Patients registered as having undergone any other type of emergency abdominal surgery were included in the non-DCS group.

### Statistical analysis

First, we analyzed the baseline characteristics of the two groups. We used Mann–Whitney U test or χ^2^ test for the analysis. Second, to obtain the potential risk factors for DCS, we performed a logistic regression analysis with DCS as the dependent variable. The independent variables were age, gender, HR, consciousness (GCS), body temperature (BT), type of injury (blunt or penetrating), ISS, and head AIS. We also categorized these potential risk factors and created a score based on the regression coefficient. To evaluate the optimal cutoff value, we subsequently performed a receiver operating characteristic (ROC) curve analysis with the proposed new score and calculated the sensitivity and specificity.

Finally, we used the data from the JTDB in 2015 to validate our results. Patient selection was performed accordingly. We performed an ROC analysis to evaluate the new score of DCS performance. All statistical analyses were performed using SPSS version 25 (SPSS for Macintosh, IBM, 2017, Chicago, IL, USA).

### Ethics

The Ethics Committee of Tokyo Medical and Dental University approved this study (#2192). The requirement for informed consent for each patient was waived on the basis of the retrospective design and the use of anonymized patient and hospital data.

## Results

### Patient selection and characteristics

**Figure 1:**
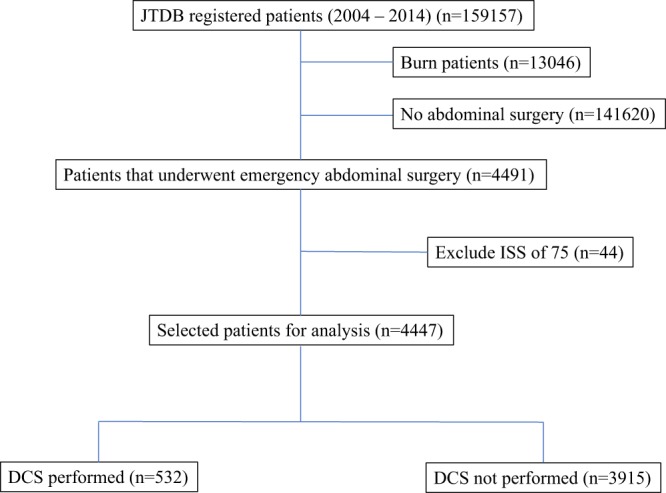
Study population. Of the 159,157 registered trauma patients, 13,046 burn victims and 44 patients with ISS of 75 were excluded. A total of 4,447 patients underwent emergency abdominal surgery, including 532 patients in the DCS group (12.0%) and 3,915 patients in the non-DCS group (88.0%).

The study population is shown in Fig. 1. Of the 159,157 registered trauma patients, 13,046 burn victims and 141,620 patients who did not have any type of abdominal surgery were excluded. Of the 4491 patients who underwent emergency abdominal surgery, 44 patients with an ISS of 75 were excluded. The 4447 patients in the study population included 3374 patients (75.9%) with blunt trauma and 1049 patients (23.6%) with penetrating trauma. Of the 4447 patients who underwent emergency abdominal surgery, 532 patients (12.0%) were included in the DCS group and 3915 patients (88.0%) were included in the non-DCS group (Fig. 1). The baseline characteristics are shown in Table 1.

**Table 1:** Baseline characteristics.

Operation	DCS	Non-DCS	P value
n	532	3915	
Age (years)	55 [33–69]	49 [31–65]	N.S.
Male, n (%)	381 (71.6)	2822 (72.1)	N.S.
Blunt Injury, n (%)	485 (91.5)	2889 (74.2)	<0.001
ISS	34 [21–43]	17 [9–29]	<0.001
AIS Head ≥ 4, n (%)	70 (13.3)	275 (7.2)	<0.001
Time to surgery, minutes	92 [61–143]	133 [85–222]	<0.001
Heart Rate, bpm	100 [80–120]	91 [76–110]	<0.001
Systolic Blood Pressure, mmHg	83 [62–110]	110 [84–132]	<0.001
GCS	12 [7–14]	14 [12–15]	<0.001
Body Temperature, °C	35.5 [34.8–36.2]	36.1 [35.4–36.7]	<0.001
FAST positive, n (%)	393 (78.6)	1963 (59.3)	<0.001
Blood Transfusion, n (%)	506 (96.6)	2470 (64.6)	<0.001
Mortality, n (%)	297 (59.0)	711 (19.7)	<0.001

Numeric values are expressed as median [25^th^–75^th^ percentiles].

Baseline characteristics of the DCS group and the non-DCS group. The DCS group had a significantly higher percentage of blunt injury, higher ISS, and higher combined injury of AIS ≥ 4 head injuries. Time to surgery was significantly shorter in the DCS group. The DCS group had significantly worse vital signs. In addition, more patients in the DCS group were FAST-positive, required more blood transfusions, and had higher mortality.

The DCS group presented a higher rate of blunt injury [485 (91.5%) vs. 2889 (74.2%); p < 0.001] and a significantly higher ISS (median 34 vs. 17; p < 0.001). The DCS group also included a higher rate of severe (AIS ≥ 4) head injuries [70 (13.3%) vs. 275 (7.2%); p < 0.001]. The DCS group also had a shorter time to surgery (median 92 vs. 133 min; p < 0.001). Vital signs on arrival were significantly worse in the DCS group, with higher HR, lower blood pressure, and lower GCS score. The DCS group also had a higher FAST-positive rate [393 (78.6%) vs. 1963 (59.3%); p < 0.001].

### Indication score for DCS

Table 2 shows the results of logistic regression analysis with DCS as the dependent variable and age, gender, HR, GCS, BT, injury type, ISS, and head AIS as independent variables. The results showed that the GCS, BT, and injury type are potential risk factors for DCS. In particular, blunt injury had the highest odds ratio of 2.180, implying that injury type is a significant factor for the implementation of DCS in our study cohort.

**Table 2:** Logistic regression analysis.

	Odds Ratio	95% confidence interval	p value
Age	1.007	1.001–1.012	0.025
Male	1.084	0.840–1.398	N.S.
HR	1.004	1.000–1.007	N.S.
GCS	0.939	0.911–0.967	<0.001
Body Temperature	0.772	0.700–0.851	<0.001
Blunt Injury	2.180	1.441–3.298	<0.001

Logistic regression analysis revealed that age, GCS, body temperature, and blunt injury are potential risk factors for DCS.

**Figure 2:**
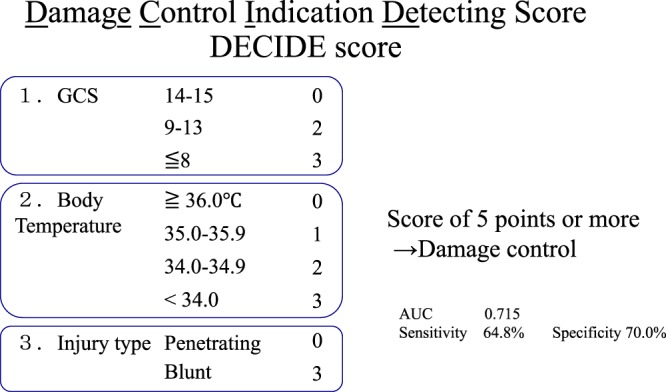
The DECIDE score. The DECIDE score consists of GCS, body temperature, and injury type, with each category being assigned three points for a maximum of nine points. Setting a threshold of five points gave the maximum AUC of 0.715, with sensitivity of 64.8% and specificity of 70.0%.

We then categorized the risk factors and repeated the logistic regression analysis. Using the acquired regression coefficient, we weighted the predictive factors and developed an indication score for DCS. We repeated the ROC analysis to calculate the most appropriate combination with the highest area under the curve (AUC). Three factors—HR, GCS, and type of injury—were implemented in our creation of the Damage Control Indication Detecting (DECIDE) score. Each category was assigned 3 points, so the highest possible score was 9 points. The ROC analysis result is shown in Fig. 2. With a cutoff value of 5 points, the AUC was 0.715, with sensitivity of 64.8% and specificity of 70.0%. Table 3 shows the cross-tabulation of the DECIDE score in the study population regarding the performance of DCS.

**Table 3:** DECIDE score and DCS in the study population.

	DCS (n = 532)	Non-DCS (n = 3915)
DECIDE score ≥ 5	345	1174
DECIDE score < 5	187	2741

This cross tabulation shows the results of the DECIDE score regarding DCS in the study population. The sensitivity was 64.8% and specificity was 70.0%.

### Decide score and mortality

**Figure 3:**
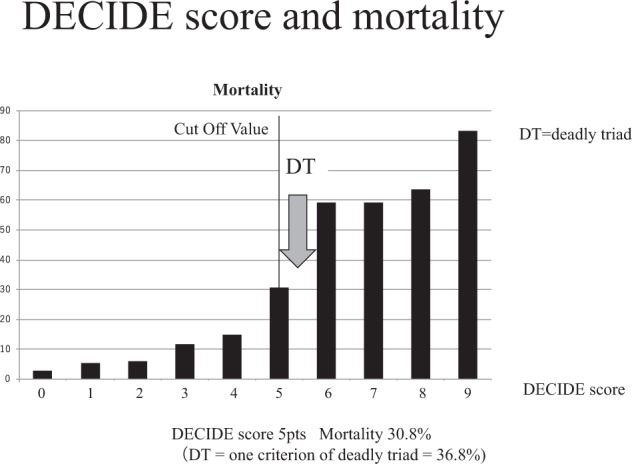
The DECIDE score and mortality. The graph shows the relationship of mortality of the DECIDE score to that of the deadly triad. The vertical line represents the cut-off value of five points. The DECIDE score of five points was associated with a mortality rate of 30.8% in our study. The gray arrow represents the mortality of the deadly triad, as one criterion of the deadly triad is said to be associated with a mortality rate of 36.8%. A positive DECIDE score of five points had a mortality rate equivalent to that of a single criterion of the deadly triad.

The relationship between the DECIDE score and mortality is shown in Fig. 3. The score was positively correlated with mortality. A DECIDE score of 4 points was associated with a mortality rate of 14.9%, whereas the rate was 30.8% for 5 points and 59.3% for 6 points. The presence of a single factor within the “deadly triad” was reported to be associated with a mortality rate of 36.8%, so our cutoff value of 5 points for the DECIDE score appeared to be legitimate because it corresponded to the mortality rate of one part of the deadly triad^6^.

### Validation of the study

**Figure 4:**
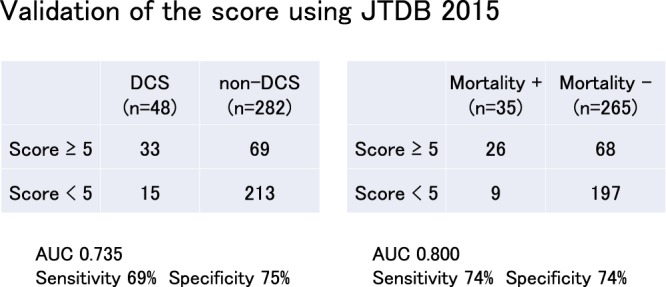
Validation of the DECIDE score using JTDB 2015. The DCS group consisted of 48 patients, whereas the non-DCS group consisted of 282 patients. The result of the ROC is as shown. The AUC was 0.735, with sensitivity of 69% and specificity of 75%. As for mortality, AUC was 0.800, with sensitivity of 74% and specificity of 74%.

Finally, we validated the proposed score using data from the JTDB in 2015 (Fig. 4). The study population consisted of 48 patients in the DCS group and 282 patients in the non-DCS group. The ROC analysis results of the performance of DCS and the DECIDE score are shown in Fig. 4. The AUC was 0.735, and upon setting a score threshold of 5 points, the sensitivity was 69% and the specificity was 75%. Furthermore, the ROC analysis for mortality was performed, with the result showing an AUC of 0.800 with sensitivity of 74% and specificity of 74%. We believe that this is a valid score to decide whether or not to perform DCS because it can be obtained simply using vital signs and the recorded mechanism of injury.

## Discussion

In the last few years, it has become clear that a paradigm shift in managing severely injured trauma patients has occurred from definitive repair to hemorrhage control, contamination containment, and initial stabilization of physiological parameters, including coagulopathy^7,8^.

There is no absolute “gold standard” for performing DCS. Building on the classical deadly triad of hypothermia (BT < 35 °C), acidosis (base deficit < −14 mmol/L), and coagulopathy (presence of medical bleeding) proposed by Morris *et al*. in 1993^9^, other researchers have set new DCS criteria.

Although one prospective study of massively transfused trauma patients in 1997 have reported the predictive model for life-threatening coagulopathy in massively transfused patients including severity of injury (ISS ≥ 25), shock (SBP < 70 mmHg and pH < 7.10), and hypothermia (BT < 34 °C)^10^, another retrospective study of exsanguinated trauma patients in 2001 has identified acidosis (pH ≤ 7.2), hypothermia (BT < 34 °C), and blood replacement >4000 mL or fluid replacement >10,000 mL as factors that predict mortality^11^. In 2010, Matsumoto *et al*., in their retrospective study of trauma patients with unstable hemodynamics after initial fluid resuscitation who had undergone DCS for severe abdominal or pelvic injuries, sought for a simplified criteria to enable rapid decision-making criteria for DCS and established the following criteria: hypotension (SBP < 90 mmHg), acidosis (base deficit < −7.5 mmol/L), and hypothermia (BT < 35.5 °C)^12^. As such, most criteria have evolved around the concept of the deadly triad.

Recently, a multicenter, retrospective study from Japan in 2016 have validated the deadly triad regarding mortality. Endo *et al*. in their report stated that the classical criteria for the deadly triad were insufficient, as they showed high specificity but inadequate sensitivity, and redefined a revised criteria of the deadly triad with fibrinolytic disorder [fibrin degradation products (FDP) > 90 μg/mL], acidosis (base deficit < −3 mmol/L), and hypothermia (BT < 36 °C)^13^.

As the trends regarding DCS criteria have emerged in recent years, they share a common characteristic. The proposed criteria often implement non-time-consuming factors that are promptly available after hospital admission, such as BT and blood gas analysis. The severity of coagulopathy is assessed directly using laboratory results, such as FDP, or indirectly by the amount of transfusion used.

Exsanguination in the first few hours of trauma is reported to be the leading cause of death^14^, and delaying surgery is a significant factor against mortality in severe trauma patients^15^. Thus, it is crucial to identify trauma patients in need of damage control resuscitation early in the course of therapeutic intervention to reduce mortality and improve outcome. Harvin *et al*. reported that the mortality of hypotensive trauma patients undergoing emergent laparotomy has not decreased in almost two decades as the rate in their report in 2017 was equivalent to that reported by Clarke *et al*. in 2002^16,17^. Hence, there is still a great need for an adequate indication for DCS that could shorten the time to surgery.

The basic assumption that the indication for DCS must be obtained promptly after arrival at a hospital using minimal examinations and time-consuming tests inspired our idea of the DECIDE score. Our score is unique in that it does not require any laboratory or mechanical data.

Consciousness, as assessed using the GCS, was a significant factor in deciding DCS in our study. Previous studies reported a decrease in the GCS as a potential risk factor associated with mortality in severe trauma patients^18^. In addition, Liao *et al*., in their review of risk factors for patients undergoing damage control laparotomy, reported that the GCS reflects initial hypoperfusion, and it could be a risk factor for a poor outcome^19^. Our score indicates that severe trauma patients who have a diminished consciousness level upon arrival are potential DCS candidates.

Our study showed that the type of injury is as critical as BT, a component of the classical deadly triad, in deciding the indication for DCS. This could be affected by the fact that most penetrating injuries in Japan are from stabbings, and gunshot wounds are rare. This could potentially explain why penetrating injuries are not fit indicators for DCS in our country, and this clearly does not apply to other nations around the world. However, our study was significant to the extent that it shows that injury type is as crucial as hypothermia in severe trauma care in Japan.

Our score is simple and can be easily used in daily trauma care to identify patients who need DCS. We believe that the indication for DCS should not be complex and overtriage should be very well accepted because the patient’s survival, not the score’s accuracy, is the sole priority.

### Limitations

There are several limitations in this study. First, this was a retrospective study rather than a randomized controlled or prospective study, which might limit the interpretation of the results. Also, not all of the facilities in Japan participate in the JTDB, and the number of participating facilities differs across the time periods that we analyzed as the number of hospitals participating in the database increased every year. There is also a possibility of regional and institutional variabilities in the quality of trauma care.

Another critical limitation is that the definition of “DCS” is not universal among the facilities registering for the database. Whether or not the performed surgery was “DCS” depended on the surgeon’s judgment. It is possible that some hospitals/surgeons define DCS as a surgery with prophylactic open abdomen, even when the patient may not have deteriorated physiologically. In addition, at some hospitals, DCS might be performed routinely, whereas at some hospitals DCS might not be performed at all. These factors could constitute a tremendous source of bias in our study and could affect the basic characteristics and severity of the analyzed patients.

In addition, about 95% of trauma cases in Japan involve blunt trauma injuries, with close to 0% of firearm injuries registered in the database. Although this percentage does not apply to our study patient cohort, the overall trend still suggests that the results of this study may not be applicable to trauma care internationally.

Moreover, the registry does not include any type of documentation regarding the massive transfusion protocol, and this could potentially limit the understanding of the baseline characteristics of the study patients.

Furthermore, this was a retrospective study covering a decade of trauma patient care in Japan. Over this period of time, certain trends regarding the criteria for DCS have emerged as mentioned earlier, leading to changes in trauma patient practice that could possibly have affected our results.

Finally, the number of patients in the validation study is small, and further validation is favorable.

## Conclusions

The need for DCS can be decided on the basis of BT, GCS, and type of injury. The score based on these variables may be used in a prehospital situation so that the trauma team can prepare for DCS even before arrival. However, trauma patients in Japan tended to have suffered blunt trauma, and firearm injuries are rare, which might imply that our results cannot necessarily be extrapolated to other nations. Further validation of this score is essential for its widespread implementation.

## Data Availability

The data analyzed during the current study (Japan Trauma Data Bank) is not publicly available as the data belongs to the Japan Trauma Care and Research, and is not made public to the third party.
